# Efficacy of the Integrative Acupuncture and Moxibustion Treatment in Patients With Major Depressive Disorder: The Study Protocol for a Multicenter, Single-Blinded, Randomized Trial in China

**DOI:** 10.3389/fmed.2022.761419

**Published:** 2022-05-30

**Authors:** Yuan Zhang, Yamin Liu, Baile Ning, Luda Yan, Lihua Wu, Delong Zhang, Changhong Li, Wenwei Ouyang, Shengyong Su, Shuo Jiang, Guangcai Zhang, Junfeng Xu, Zhen Wang, Zhong Zheng, Dong Zheng, Shan Chen, Lu Sun, Wenbin Fu

**Affiliations:** ^1^Department of Acupuncture and Moxibustion, The Second Affiliated Hospital of Guangzhou University of Chinese Medicine, Guangzhou, China; ^2^Shenzhen Bao’an Traditional Chinese Medicine Hospital, Guangzhou University of Chinese Medicine, Shenzhen, China; ^3^School of Psychology, South China Normal University, Guangzhou, China; ^4^College of Teacher Education, Guangdong University of Education, Guangzhou, China; ^5^Key Unit of Methodology in Clinical Research, The Second Affiliated Hospital of Guangzhou University of Chinese Medicine, Guangzhou, China; ^6^The First Affiliated Hospital of Guangxi Chinese Medical University, Nanning, China; ^7^The First Affiliated Hospital of Zhejiang Chinese Medical University, Hangzhou, China; ^8^Hainan Provincial Hospital of Chinese Medicine, Haikou, China; ^9^The First Affiliated Hospital of Tianjin Chinese Medical University, Tianjin, China; ^10^The Second Affiliated Hospital of Anhui Chinese Medical University, Hefei, China; ^11^Sleep Medical Center, West China Hospital of Sichuan University, Chengdu, China; ^12^Brain Hospital Affiliated Guangzhou Medical University, Guangzhou, China; ^13^Department of Psychosomatic Medicine, Guangdong Provincial Hospital of Chinese Medicine, Guangzhou, China

**Keywords:** acupuncture, moxibustion, auricular acupuncture, integrative treatment, depressive disorders, sertraline

## Abstract

**Introduction:**

Antidepressants are the front-line treatments for major depressive disorder (MDD), but remain unsatisfactory in outcome. An increasing number of patients are interested in acupuncture and moxibustion treatment as complementary therapies. This study aims to evaluate the efficacy and safety of integrative acupuncture and moxibustion (iAM) treatment in patients with MDD.

**Methods and Analysis:**

This multicenter, single-blind, 2 × 2 factorial randomized trial will enroll 592 patients with MDD of moderate severity from nine hospitals. All patients will be randomized, in a ratio of 2:2:2:1, through a computerized central randomization system, into four groups (the combined, iAM-only, sertraline-only, and placebo groups). Participants will undergo a 12-week intervention with either 50 mg of sertraline or a placebo once a day and active/sham iAM treatment three times per week. The primary outcome is depression severity, assessed using the Hamilton Depression Scale-17. The secondary outcomes include self-rated depression severity, anxiety, and sleep quality. The primary and secondary outcomes will be measured at weeks 0, 4, 8, 12, and the 8th week posttreatment. Safety will be evaluated through liver and kidney function tests conducted before and after treatment and through monitoring of daily adverse events. An intent-to-treat principle will be followed for the outcome analyses.

**Conclusion:**

This trial will provide sufficient evidence to ascertain whether iAM is effective and safe for treating MDD and provides a suitable combination strategy for treating MDD.

**Clinical Trial Registration:**

[www.chictr.org.cn], identifier [ChiCTR2100042841].

## Introduction

Depressive disorders are common worldwide, and their prevalence reaches up to 4.4 and 4.2% of the global and Chinese populations, respectively (1). Major depressive disorder (MDD) is the most prevalent subtype and a leading cause of global disease burden (2). Estimates show that by 2030, this disorder is likely to rank high in the measure of total global disability-adjusted life years (DALY) (3). If left untreated, MDD can lead to suicide in 2.5% of patients (4). Thus, it is recognized as a major public health issue, having a substantial impact on individuals, families, and society (5, 6).

MDD is characterized by a lasting dysphoric mood, loss of interest or enjoyment, as well as fatigue or reduced energy. Patients with MDD often experience anxiety symptoms, sleep disturbances, cognitive impairment, and even other somatic symptoms (7–9). The treatment is currently based on symptom control, achieving remission, and restoring patients’ function to baseline levels (10). The guideline-recommended treatments include a range of psychological interventions, such as behavioral activation, cognitive behavioral therapy, counseling, interpersonal psychotherapy, and pharmacological interventions such as first- and second-generation antidepressants. Despite the vast range of available treatments, a survey based on 17 countries has found that untreated rates of mental disorders are very high in low- and middle-income countries, ranging from 76 to 85% (11). A Chinese report illustrated that the number of patients with depressive symptoms, who received treatment for MDD was < 10% in four provinces (12).

The reasons for low treatment rates are inadequate medical resources, the social stigma associated with mental disorders, concerns about adverse drug reactions, and over-reliance on medication (12). These factors adversely affect patients, minimize their chances and motivation to receive any treatment, and are barriers to treatment. Due to the lack of professional psychologists, particularly in low- and middle-income countries, antidepressants are more frequently prescribed than psychological interventions (13). Second-generation antidepressants, such as selective serotonin reuptake inhibitors, are usually recommended as the front-line treatment for MDD based on the clinical guidelines (13–15). However, there are still debates regarding the effectiveness of medication because of limitations, such as delayed onset (16, 17), inadequate response in up to 50% of patients (4, 18), and lack of adherence in 30% (19). These limitations are additional barriers to effective treatment. Interestingly, patients nowadays show a preference for complementary therapies (12, 20).

Acupuncture is a therapy representative of Traditional Chinese Medicine (TCM) and is a common clinical practice in China (21). Internationally, acupuncture is used to treat depression and is recommended for primary care in the United Kingdom (22, 23). In 2016, the American College of Physicians recommended it as a complementary and alternative medicine in the clinical practice guidelines for adult patients with MDD (15). Currently, various styles of acupuncture are used in clinical practice, ranging from traditional/classical to modern, such as abdominal, auricular, and electro-acupuncture. The advantages of these different styles may vary, and two or more techniques may be applied jointly in clinical practice for diseases with complex symptoms (24). Moxibustion is another treatment that involves burning moxa and producing heat to stimulate acupoints and is mostly used with acupuncture to enhance the treatment’s effect (25). Although MDD is not a simple disease consisting of a single symptom, most previous trials on treatment and acupuncture have only focused on the outcomes of a single-acupuncture therapy, which is deficient in terms of integrative intensity and treatment principles. Although there is an increasing accumulation of randomized trials on acupuncture and depression, these studies have methodological limitations and do not provide high-quality experimental evidence (26).

Since 2000, we have studied the effect of acupuncture on depression and formed an integrative acupuncture and moxibustion (iAM) treatment protocol called “Shugan Tiaoshen” (27). Specifically, this treatment protocol uses acupuncture and moxibustion to regulate the “Liver Qi” and spirit, which are associated with managing mood and energy in TCM theory. Our previous pilot studies (28, 29) showed that this integrative therapy could improve depressive symptoms and sleep quality in people with mild to moderate depression. Therefore, this protocol was designed as a large-sample multicenter 2 × 2 factorial randomized trial, which aimed to investigate the effectiveness and safety of this integrative therapy for patients with moderate MDD, as well as identify a suitable treatment strategy.

## Materials and Methods

### Design and Setting

This study is being conducted at nine clinical hospitals in China, covering the northern, southern, eastern, and western parts of the country. The primary sponsor is the Second Affiliated Hospital of Guangzhou Chinese Medical University (Guangdong Provincial Hospital of Chinese Medicine, GPHCM). Ethical approval was obtained from the Ethics Committee of the GPHCM (No. BF2020-186). This trial was registered at the Chinese Clinical Trial Registry (ChiCTR2100042841), and all patients will be required to provide written informed consent to participate. The study is being conducted in compliance with the local regulations and international principles established in the Declaration of Helsinki. This protocol follows the guidelines of the Standards for Reporting Interventions in Clinical Trials of Acupuncture (30) and the Standard Protocol Items: Recommendations for Interventional Trials (31).

A total of 592 patients with MDD with moderate symptoms will potentially be recruited and randomly assigned per a 2 (active/sham iAM) × 2 (sertraline/placebo) factorial design with a ratio of 2:2:2:1 into active iAM and sertraline (hereafter referred to as combined treatment group), active iAM and placebo (iAM-only group), sham iAM and sertraline (sertraline-only group), and sham iAM and placebo (placebo group).

### Eligibility Criteria

The inclusion criteria for this study are as follows: (1) diagnosis of MDD according to the Diagnostic and Statistical Manual of Mental Disorders 5th Edition criteria and the Standard for TCM Diseases and Syndromes Therapeutic Results, published by the Chinese TCM Authority (ZY/T001.1-94); (2) age of 18–65 years, with an educational level higher than junior middle school; and (3) with a written consent form. Patientes will be exclude if they meet the following criteria: (1) Hamilton Depression Scale-17 (HAMD-17) scores ≤17 or >24; (2) suicidal tendency; (3) treatment with antidepressants over the last 6 weeks before enrolment; (4) history of psychiatric diseases (schizophrenia, bipolar disorder, substance abuse, and others); (5) brain organic diseases, severe somatic diseases, patient or strong family history of epilepsy; (6) skin lesions or diseases, severe diabetes, tumor, and significant organ dysfunction or severe internal diseases; (7) pregnant, intending to become pregnant, or lactating.

### Recruitment and Consent

**FIGURE 1:**
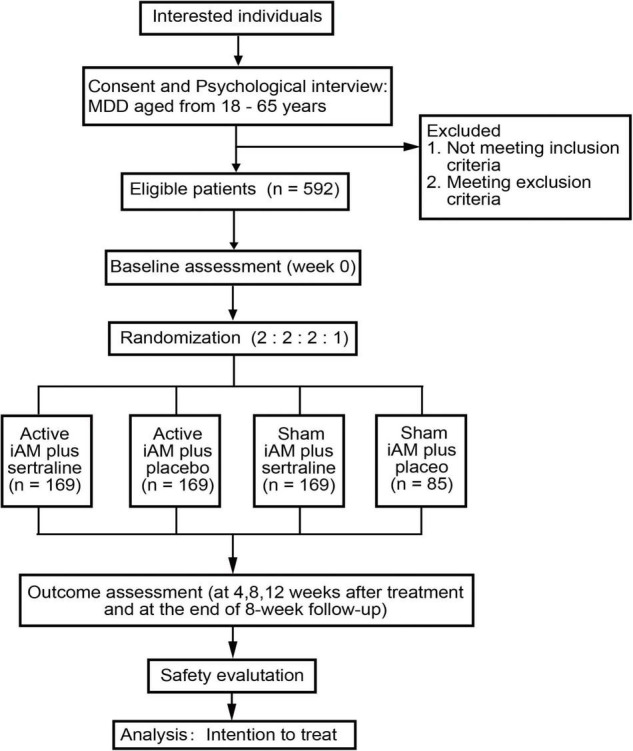
Randomized trial flow diagram.

Participants will be recruited from the nine centers via local advertising, newspapers, and the internet, and interested individuals will be instructed to contact research assistants by phone or email to make an appointment. During the first visit, psychological interviews will be conducted, evaluated, and recorded according to the inclusion and exclusion criteria to ensure each individual’s eligibility. Eligible participants will be given detailed information about the trial and consent forms. Then, research assistants will obtain a signed form from those willing to participate. The study flow diagram is shown in Figure 1.

### Randomization and Allocation

The allocation will be concealed using a central randomization method. A 2:2:2:1 permuted block sequence will be generated by the central randomization system of the GPHCM, which divides the eligible participants into either the combined treatment, iAM-only, sertraline-only, or placebo groups. The entire allocation process will be performed by a specific staff member.

### Blinding

The participants, data managers, and statisticians will be blinded to the treatment allocations, which will not be revealed until the end of the study. However, this cannot be done for acupuncturists, because acupuncturists must provide the proper iAM treatment, active or sham, according to the patients’ allocations. Participants will be given the corresponding labeled bottle filled with either medication or placebo pills, which will be identical in appearance. Each participant will be treated with acupuncture and moxibustion in a separate room to ensure adequate privacy. Due to the nature of auricular acupuncture, acupuncturists rather than participants will be instructed to remove the needles. In addition, acupuncturists and researchers will be instructed not to communicate with the participants about the possibility of their allocations.

### Intervention

**FIGURE 2:**
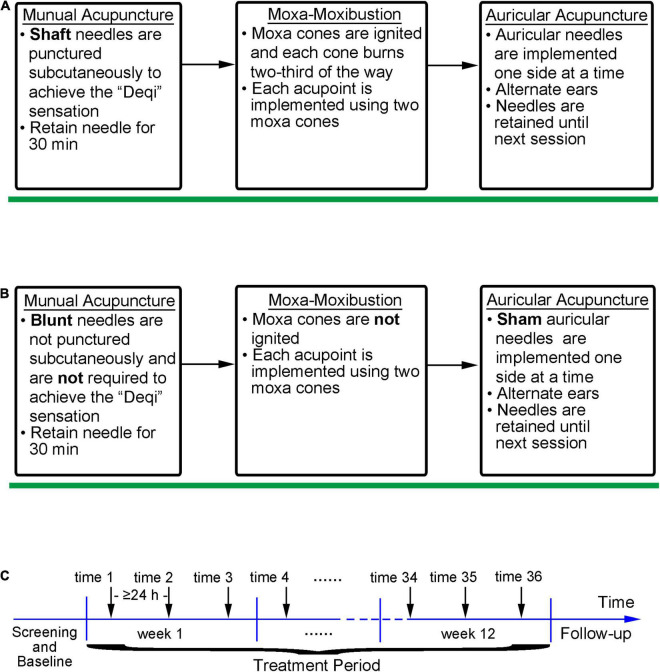
The iAM implementation and intervention processes. **(A)** Implementation process of active iAM intervention; **(B)** Implementation process of sham iAM intervention; **(C)** Intervention frequency of the iAM.

The participants will receive active/sham integrative acupuncture and moxibustion treatment three times a week, with each interval > 24 h, for a total of 36 times for 12 consecutive weeks. The integrative treatment process is illustrated in Figure 2.

#### The Integrative Acupuncture-Moxibustion Treatment and Sham Method

**FIGURE 3:**
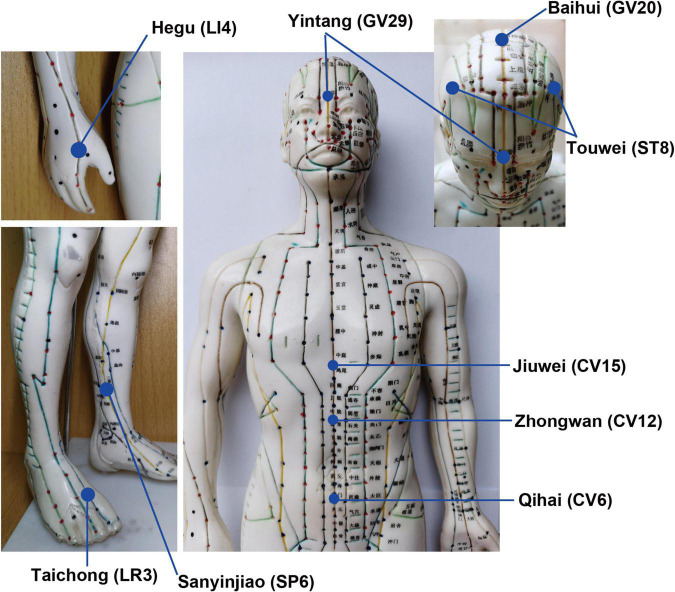
Acupuncture acupoints and locations.

**FIGURE 4:**
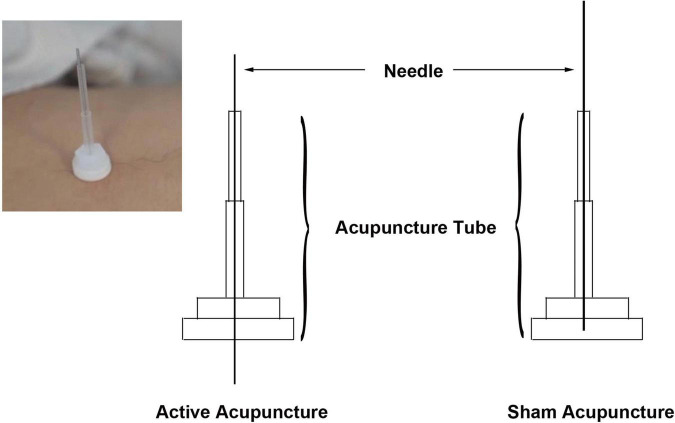
Active vs. sham acupuncture. Shaft needles are punctured subcutaneously in active acupuncture, while blunt needles are not punctured subcutaneously in sham acupuncture.

The iAM treatment protocol comprises three interventions: manual acupuncture, moxibustion, and auricular acupuncture (Figure 2). For the manual acupuncture, patients will be asked to lie in the supine position, wearing eye masks for a better curative effect. After skin disinfection with 75% alcohol, acupuncture will be performed at the following acupoints: Baihui (GV20), Yintang (GV29), Touwei (ST8), Jiuwei (CV15), Zhongwan (CV12), Qihai (CV6), Taichong (LR3), Hegu (LI4), and Sanyinjiao (SP6) using tube needles (Hwato Suzhou Medical Instruments, Suzhou, China). The locations of the acupoints are shown in Figure 3. Needle lifting, thrusting, and twisting will be used to achieve the “Deqi” sensation, which is believed to indicate effective needling based on the TCM theory (32). The needles will be retained *in situ* for 30 min and then removed. The same procedure will be performed for the sham conditions, but without percutaneous puncture using blunt-tipped needles, meaning that the “Deqi” sensation will not be achieved. Detailed information about the active/sham acupuncture method and needle sizes are shown in Table 1 and Figure 4.

**TABLE 1:** Details regarding active acupuncture and sham methods.

No.	Acupoint	Active acupuncture		Sham acupuncture	
		Needing method	Needles sizes	Needing method	Needle size
1	Baihui (GV20)	Punctured perpendicularly reaching the periosteum	0.25 × 25 mm	All needle slightly pressed but did not penetrate the skin	0.40 × 25 mm
2	Yintang (GV29)	Punctured perpendicularly reaching the periosteum	0.25 × 25 mm		0.40 × 25 mm
3	Touwei (ST8)	Punctured perpendicularly reaching the periosteum	0.25 × 25 mm		0.40 × 25 mm
4	Jiuwei (CV15)	Punctured perpendicularly 0.3 cun	0.22 × 40 mm		0.40 × 40 mm
5	Zhongwan (CV12)	Punctured perpendicularly 1 cun	0.22 × 40 mm		0.40 × 40 mm
6	Qihai (CV6)	Punctured perpendicularly 1 cun	0.22 × 40 mm		0.40 × 40 mm
7	Taichong (LR3)	Punctured perpendicularly 0.5 cun	0.25 × 25 mm		0.40 × 25 mm
8	Hegu (LI4)	Punctured perpendicularly 0.5 cun	0.25 × 25 mm		0.40 × 25 mm
9	Sanyinjiao (SP6)	Punctured perpendicularly 0.5 cun	0.25 × 25 mm		0.40 × 25 mm

*1 cun = 25 mm.*

**FIGURE 5:**
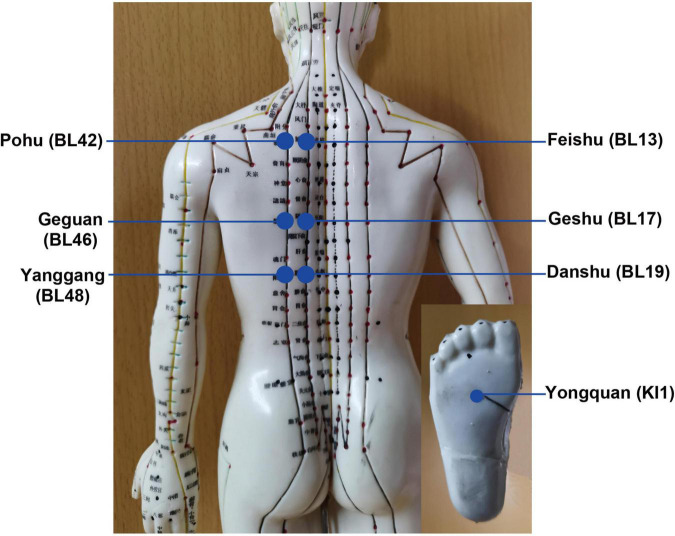
Moxibustion acupoints and locations.

**FIGURE 6:**
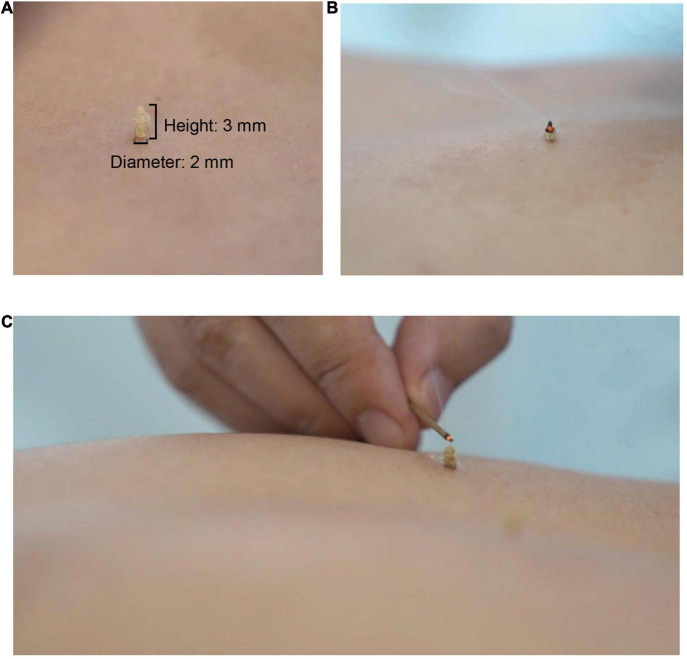
Moxibustion intervention. **(A)** Presents the size of a moxa cone; **(B)** and **(C)** present the demonstration of lighting a moxa cone.

After acupuncture, moxibustion will be performed (Figure 2). Patients will be asked to lie in the prostrate position, wearing eye masks. The alternate groups of selected acupoints are Feishu (BL13), Geshu (BL17), Danshu (BL19), and Yongquan (KI1) in one group, and Pohu (BL42), Geguan (BL46), Yanggang (BL48), and Yongquan (KI1) in the other (Figure 5). Wanhua oil will be smeared evenly on the skin to fix moxa cones and prevent heating. A moxa cone, 2 mm in diameter and 3 mm in height, will be placed and ignited using a joss stick (Figure 6). It will be removed when two-thirds of it have burned off. Two moxa cones will be applied at each point. In sham moxibustion, the moxa cones will not be ignited.

**FIGURE 7:**
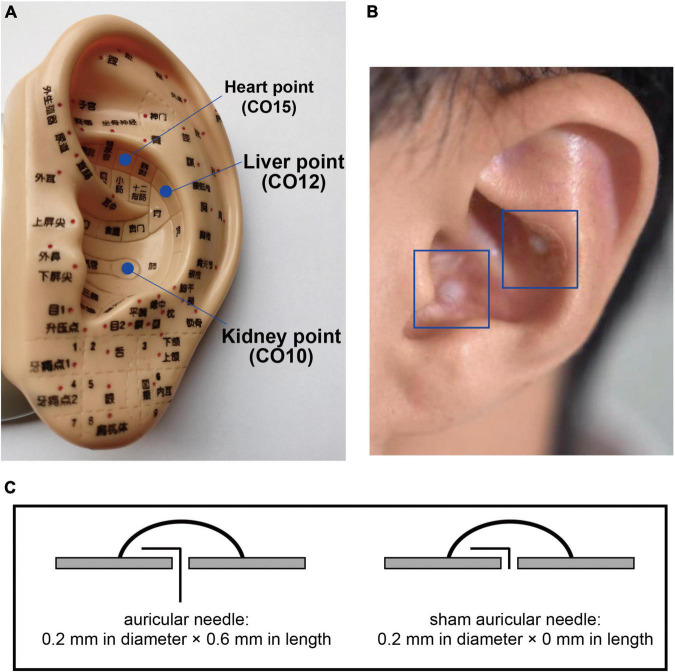
Auricular points and needles. The locations of selected auricular points are shown in **(A)**; the intervention methods of the auricular needles and sham needles are shown in **(B)** and **(C)**.

Finally, auricular acupuncture will be performed. The acupoints of the heart (CO15), liver (CO12), and kidney (CO10) will be selected for the intervention (Figure 7A). Auricular needles (33) (0.2 mm in diameter, 0.6 mm in length; Seirin Co Ltd., Shimize-City, Japan) will be used on the points of the unilateral auricle and removed by the acupuncturists in the subsequent treatment. Auricular acupuncture will be conducted on one side of the auricle for one treatment and the other side for the next treatment, alternating between the two sides of auricles. For sham conditions, fake auricular needles will be used and not placed (Figures 7B,C).

The participants in the combined treatment and the iAM-only groups will receive the iAM treatment, while in sertraline-only and placebo group will receive the sham iAM treatment.

#### Treatment Vs. Placebo

The participants in the combined treatment and sertraline-only groups will be given a bottle filled with the antidepressant medication sertraline (Approval No. h10980141, Pfizer Co., Ltd.), and instructed to take 50 mg orally after breakfast, once a day, continuously for 12 weeks. The participants in the iAM-only and placebo groups will receive a bottle filled with placebo pills made of starch instead. The placebo pills do not differ from the antidepressants in appearance.

Due to changes in the participants’ illness, there will be no prohibitions on medication changes during the trial. However, any changes will be checked by psychiatrists and recorded.

### Study Visits and Measures

Five visits will be performed in this study. The study schedules and measures are displayed in Table 2. During the screening visit, patients will undergo psychological interviews to screen for eligibility according to the inclusion and exclusion criteria. Diagnosis of TCM syndrome types, demographic information, and medical history will be obtained. Eligible patients will then undergo assessment and baseline data collection. Three visits will take place during the treatment period (weeks 4, 8, and 12), with one follow-up visit at week 20. The study assessments will include HAMD-17, the Patient Health Questionnaire-9 (PHQ-9), the Hamilton Anxiety Scale (HAMA), the Pittsburgh Sleep Quality Index (PSQI), sleep actigraphy, and adverse event reporting. Liver and kidney function tests will also be performed to assess safety, and blood samples will be collected only at baseline (week 0) and after treatment (week 12). Data collection and analysis will be conducted by three research assistants blinded to the treatment allocations.

**TABLE 2:** Schedule of study visits and assessments during the enrolment, treatment, and follow-up periods.

		Treatment period			Follow-up
			
	Screening and baseline	Week 4	Week 8	Week 12	Week 20
Inclusion/exclusion criteria	×				
Consent	×				
Allocation	×				
Active/Sham iAM intervention		×	×	×	
Sertraline/placebo drug intervention		×	×	×	
Demographic information	×				
Medical history taking	×				
TCM Syndrome Types	×				
PHQ-9	×	×	×	×	×
HAMD-17	×	×	×	×	×
HAMA	×	×	×	×	×
PSQI	×	×	×	×	×
Sleep Actigraphy measures	×	×	×	×	×
Blood sample for liver and kidney function tests	×				×
Adverse events report		×	×	×	×
Adherence scale		×	×	×	×
Medication record		×	×	×	×

*iAM, integrative acupuncture and moxibustion; TCM, Traditional Chinese Medicine; PHQ-9, Patient Health Questionnaire; HAMD-17, Hamilton Depression Scale-17; HAMA, Hamilton Anxiety Scale; PSQI, Pittsburgh Sleep Quality Index; ×, required item.*

### Outcome Measures

#### Primary Outcomes

The HAMD-17 is an established clinician-rated assessment of depressive symptom severity and encompasses psychological and somatic symptoms (34). It assesses the severity of 17 depression symptom items over the previous week and has five factors: anxiety/somatization, cognitive impairment, “psychomotor retardation,” weight, and sleep changes. A higher HAMD-17 score implies more severe depressive symptoms. Severity is classified as no depression (score ≤ 7), mild (score 8–16), moderate (score 17–24), and severe (score ≥ 25). A score of <8 will be interpreted as remission, and a decrease greater than 50% as an effective treatment response. This measure has been shown to be reliable (35, 36). And in this study, the HAMD-17 scores measured in week 12 after treatment are defined as the primary outcome.

#### Secondary Outcomes

The PHQ-9 is a self-administered version of the Primary Care Evaluation of Mental Disorders diagnostic instrument for depression disorders (37). Its score for each criterion ranges from 0 (“not at all”) to 3 (“nearly every day”) (37). Thus, the higher the score, the more severe the patient’s depression. It is sensitive to changes and is used as a brief depression severity measurement (38, 39). The 14-item version of the HAMA is designed to standardize clinical assessments of anxiety, where the scores for each item are rated on a 5-point scale from 0 (“not at all”) to 4 (“nearly every day”). HAMA-14 classifies anxiety factors into physical and mental symptoms. Items 1–6 and 14 reflect mental symptoms, while items 7–13 reflect somatic anxiety symptoms.

The PSQI is a self-rated questionnaire that assesses sleep quality and disturbances for up to a 1-month period. It is a recommended measure for treatment effectiveness studies of global sleep quality. From the 19 included items, seven sub-scores will be calculated, including sleep quality, latency, habitual efficiency, duration, disturbances, medication use, and daytime dysfunction (40). The sum of these components yields a global score of 21, with a higher score indicating poorer sleep quality. A PSQI score > 5 implies poor sleep, and a three-point change suggests a clinical effect. In addition, actigraphy, which used a bracelet-like monitor, will be used as a supplemental tool to assess sleep quality (YWK-P9; BOZHILUN, Inc., Shenzhen, Guangdong, China). It can monitor data regarding real-time sleep quality, blood oxygen saturation, blood pressure, heart rate, exercise situation, and other indicators, which can be used to assess the physical condition. In our study, we will use this method to collect quantitative sleep data and supplement sleep quantity information. Each participant will have a smartphone application connected by Bluetooth to the actigraphy to collect data.

#### Safety Assessment

Venous blood samples will be collected through venipuncture to assess liver and kidney function at baseline and posttreatment. Adverse event records will be monitored and recorded at every visit to assess the safety of the intervention.

### Quality Control

Before the start of the recruitment period, a training workshop will be organized for the entire research team, including acupuncturists and research assistants. Acupuncturists who will provide the treatment are licensed by the Ministry of Health of the People’s Republic of China and will have received training in the application of integrative acupuncture and moxibustion treatment. These requirements will be ensured before the trial to ensure strict adherence to the study protocol and familiarity with the administration process. The research team will also be supplied with a written protocol and standard operating procedure documents.

Data will be collected from information recorded in the case report forms and an online data management platform (eMedInform, LinkerMedTechCo., Ltd., Beijing, China), a data recording and control system for clinical scientific research. All modifications will be marked on the case report forms and system. Data quality will be checked regularly by research assistants, which will be overseen by monitors. Audits will be performed regularly by the GPHCM Department of Science Research. Data monitoring will be conducted regularly, adhering to standard operating procedures by the Guangdong International Clinical Research Center of Chinese Medicine (Guangzhou, China). In case of participant withdrawal during the treatment period or the follow-up phase, the reasons shall be clarified only if the participant is willing and the rate statistically analyzed.

### Statistical Analysis

Efficacy analyses will be performed based on the intent-to-treat (ITT) principle unless stated otherwise. We have defined two study populations: full analysis set (FAS) and per-protocol set (PPS). The FAS is defined as all treated patients with a baseline HAMD-17 score and at least one HAMD-17 score on treatment. PPS includes only those patients who all completed the allocated treatment. Demographic and other basic characteristics will be summarized according to the assigned groups. Categorical variables will be summarized as frequencies and percentages, and continuous variables as mean ± SD for normally distributed and median for non-normally distributed data.

Two separate analyses of the primary outcomes will be performed. First, for the long-term effect, an analysis of variance model with a 95% confidence interval will be used to compare the mean change in the HAMD score among the groups without adjusting for any covariates. Second, a linear mixed model will be performed. For the secondary outcomes, the chi-square test will be used to assess the effect of the intervention on binary outcomes. Logistic regression analysis or generalized estimating equations will be used to analyze the categorical outcomes. The reliability, validity, and item responses of the questionnaires will also be included in our analyses. Safety analyses will be performed on safety set (SS), i.e., all randomized participants administered at least one treatment. All statistical analyses will be performed using SAS 9.3 or SPSS 18.0. All tests of significance will be two-sided, with a maximal type I error risk of 5%.

### Sample Size Calculation

The sample size was calculated based on the mean and SD of the HAMD-17. According to our previous pilot study, the data measured after treatment for 8 weeks can be considered clinically significant (10.47 ± 6.24 in the combined treatment, 12.03 ± 5.78 in the iAM-only, 12.17 ± 5.78 in the sertraline-only, and 19.11 ± 3.92 in the placebo groups). PASS 11.0 was applied to calculate sample size. We applied a 2:2:2:1 ratio to the four groups. For a statistical power of 90%, with a two-tailed significance level of 5%, the sample size was 72 for the placebo group and 144 for each of the other three groups (alpha = 0.05, beta = 0.10, *n* = 503). Assuming a dropout rate of approximately 15%, we required a sample size of 85 for the placebo group and 169 for each of the remaining three groups.

## Discussion

MDD is a prevalent disorder with a low treatment rate and significant burden (12). Pharmaceutical therapies are the guideline-recommended front-line treatments (15, 41), but 30–50% of patients with MDD do not respond to antidepressant medications (4). Moreover, the side effects of these treatments often result in low adherence rates and limited efficacy. As traditional Chinese therapies, acupuncture and moxibustion are popular and widely accepted. An increasing number of studies in the literature have demonstrated that acupuncture and related treatments may be safe and effective for depressive disorders (42, 43).

Although the antidepressant qualities of acupuncture are widely recognized, a single-acupuncture technique can not completely treat the clinical issue due to the complexity and diversity of depressive symptoms. We formulated iAM treatments to improve the efficiency of single-acupuncture therapy. The acupoint selection protocol was based on the TCM theory of regulating “Liver Qi” and spirit, which, according to this theory, play essential roles in managing mood and energy. If “Liver Qi” stagnates, it may negatively affect mood and result in apathy and low self-esteem (29). The integrative treatment process is formulated in three steps; the first step applies acupuncture to regulate “Liver Qi” and spirit, the second applies moxibustion to augment the effect, and the third applies auricular acupuncture to consolidate and extend the curative effect.

To our knowledge, this study is the first multicenter, large-sample, 2 × 2 factorial randomized trial to date assessing the efficacy and safety of iAM treatment in patients with MDD. In this regard, this will be the most comprehensive nationwide trial in terms of the participants, as they will be recruited from nine clinical centers covering seven provinces and eight cities in China. In this four-armed clinical trial, we will focus on the different efficiencies among active iAM plus antidepressants, active iAM plus placebo, sham iAM plus antidepressants, and sham iAM plus placebo to treat MDD. We will then investigate whether iAM positively affects MDD, as well as any augmentative effects and safety concerns when combined with sertraline. Given these outcomes, we can assume that this treatment program could play an essential role in preventing depression. In addition, this study is also expected to form a standardized, applicable integrative regimen to promote extensive implementation in improving depressive disorders.

This study has two limitations. Due to the nature of intervention in clinical trials, the study cannot be conducted in an ideal blind-controlled setting. Moreover, the outcomes of our study cannot be interpreted as effective for any one of these three therapies in iAM treatments when used separately.

## Conclusion

This clinical trial is designed to prove the hypothesis that integrative acupuncture and moxibustion treatment can augment the effectiveness and safety of conventional antidepressant therapy. The results will also provide more evidence regarding the effectiveness and safety of integrative acupuncture and moxibustion, potentially leading to alterations or supplementations to the routine treatment strategy.

## Ethics Statement

The studies involving human participants were reviewed and approved by the Ethics Committee of The Second Affiliated Hospital of Guangzhou Chinese Medical University (No. BF2020-186). The patients/participants provided their written informed consent to participate in this study. Written informed consent was obtained from the individual(s) for the publication of any potentially identifiable images or data included in this article.

## Author Contributions

YZ, BN, LS, and WF designed the study protocol. YL, LY, LW, and DZ assisted in the development and operationalization of the study methods. YZ, YL, BN, LY, LW, and DZ wrote the grant proposal. YZ and YL drafted the manuscript. YZ, YL, BN, LY, and LS participated in coordinating the study. CL and DZ assisted with data collection activities. WO generated the random allocation sequences for randomization and was the trial statistician. SS, SJ, GZ, JX, ZW, ZZ, DZ, and SC developed and delivered study implementation strategies in collaboration with YZ, YL, and LW. All authors critically reviewed and approved the final manuscript.

## Conflict of Interest

The authors declare that the research was conducted in the absence of any commercial or financial relationships that could be construed as a potential conflict of interest.

## Publisher’s Note

All claims expressed in this article are solely those of the authors and do not necessarily represent those of their affiliated organizations, or those of the publisher, the editors and the reviewers. Any product that may be evaluated in this article, or claim that may be made by its manufacturer, is not guaranteed or endorsed by the publisher.
